# THE BLUES AND OLDER MINORITY MUSICIANS: MORE THAN JUST MUSIC XXXI

**DOI:** 10.1093/geroni/igae098.2243

**Published:** 2024-12-31

**Authors:** Michael Marcus

**Affiliations:** University of Maryland Baltimore County, Raleigh, North Carolina, United States

## Abstract

This session has been a popular academic and social event since 1993. We’re on a mission from God.

